# Direct and indirect effects of fibroblast growth factor 23 on the heart

**DOI:** 10.3389/fendo.2023.1059179

**Published:** 2023-02-24

**Authors:** Toshiaki Nakano, Hiroshi Kishimoto, Masanori Tokumoto

**Affiliations:** ^1^ Center for Cohort Studies, Graduate School of Medical Sciences, Kyushu University, Fukuoka, Japan; ^2^ Department of Medicine and Clinical Science, Graduate School of Medical Sciences, Kyushu University, Fukuoka, Japan; ^3^ Department of Nephrology, Fukuoka Red Cross Hospital, Fukuoka, Japan

**Keywords:** FGF23, Heart, Left ventricle hypertrophy, FGFR4, cardiac event

## Abstract

Fibroblast growth factor (FGF)23 is a bone-derived phosphotropic hormone that regulates phosphate and mineral homeostasis. Recent studies have provided evidence that a high plasma concentration of FGF23 is associated with cardiac disease, including left ventricular hypertrophy (LVH), heart failure, atrial fibrillation, and cardiac death. Experimental studies have shown that FGF23 activates fibroblast growth factor receptor 4 (FGFR4)/phospholipase Cγ/calcineurin/nuclear factor of activated T-cells signaling in cardiomyocytes and induces cardiac hypertrophy in rodents. Activation of FGFR4 by FGF23 normally requires the co-receptor α-klotho, and klotho-independent signaling occurs only under conditions characterized by extremely high FGF23 concentrations. Recent studies have demonstrated that FGF23 activates the renin-angiotensin-aldosterone system (RAAS) and induces LVH, at least in part as a result of lower vitamin D activation. Moreover, crosstalk between FGF23 and RAAS results in the induction of cardiac hypertrophy and fibrosis. In this review, we summarize the results of studies regarding the relationships between FGF23 and cardiac events, and describe the potential direct and indirect mechanisms whereby FGF23 induces LVH.

## 1 Structure and function of fibroblast growth factor 23

Fibroblast growth factors (FGFs) are polypeptide growth factors with a broad range of biological functions, including the regulation of embryonic development, organogenesis, metabolism, angiogenesis, mitogenesis, and cellular differentiation (1, 2). The FGF family comprises 22 members, and FGF 23 belongs to the FGF 19 subfamily, and because this a circulating hormone, it is termed endocrine FGF (3). FGF23 is a 251-amino acid protein and is principally synthesized by osteoblasts and osteocytes. The classical target organs of FGF23 are the kidney and parathyroid glands, and FGF23 has its physiological effects through fibroblast growth factor receptors (FGFRs)1, 2, and 4, with α-klotho acting as a co-factor (4). The C-terminus of FGF23 contains the binding site for α-klotho and the N-terminus contains the binding site for FGFRs. The O-linked proprotein convertase cleavage site is stabilized through O-linked glycosylation by N-acetylgalactosaminyltransferase 3 (GALNT3), which protects FGF23 against proteolytic cleavage (5). FGF23 acts on the kidney *via* FGFR1c/α-klotho-mediated signaling to regulate phosphate excretion and vitamin D metabolism. Dietary phosphate intake stimulates the production and secretion of FGF23 by osteocytes, and FGF23 reduces phosphate reabsorption by reducing the expression of the sodium/phosphate co-transporters NaPi-2a and NaPi-2c in the proximal tubules of the kidney (6). In addition, FGF23 reduces the synthesis of active vitamin D by downregulating 1α-hydroxylase (CYP27B1) and upregulating 24-hydroxylase (CYP27B1) (7). FGF23 also inhibits the secretion of parathyroid hormone (PTH) by the parathyroid gland (8). It is normally secreted by osteocytes, but under pathological conditions, FGF23 can be secreted by the heart (9–14), liver (15), kidney (16), macrophages (17), or bone marrow (18).

## 2 FGF23 and cardiac events

Cardiovascular disease is the leading cause of mortality worldwide and is highly prevalent in the general population (19). In 2008, the circulating FGF23 concentrations of patients undergoing hemodialysis were reported to be associated with mortality for the first time (20). Since then, a large number of clinical studies have shown that high plasma concentrations of FGF23 are associated with left ventricular hypertrophy (LVH), heart failure, and mortality in the general population, and especially in patients with chronic kidney disease (CKD). In the present review, we summarize the published clinical evidence regarding the relationships between FGF23 and cardiac events and then discuss the effects of FGF23 on the heart.

### 2.1 FGF23 and LVH

Several previous studies have shown an association between high circulating concentrations of FGF23 and a high risk of LVH in the general population (21–24) and patients (25–32). In addition, there is a particularly strong association in patients with CKD (25, 26, 28, 30, 32). High circulating FGF23 concentrations are associated with concentric hypertrophy rather than eccentric hypertrophy (21, 30). These findings suggest that FGF23 might increase ventricular wall thickness. Finally, a recent clinical study showed that etelcalcetide reduces the circulating concentration of FGF23 and inhibits the progression of LVH (33). In this study, the concentration of FGF23, but not the levels of renin-angiotensin-aldosterone system (RAAS)-related parameters, showed a clear association with left ventricular mass index (34).

### 2.2 FGF23 and heart failure

Previous observational studies have demonstrated that the circulating concentration of FGF23 is related to heart failure (HF) (22, 29, 35–45), and this association appears to be stronger in patients with CKD (36, 37, 44, 46), but is not affected by adjustment for kidney function (22, 35, 37, 39, 41–44). Some previous studies have shown significant relationships between high circulating FGF23 concentration and low ejection fraction (27, 28, 45, 47–49). In addition, high FGF23 concentration was shown to be associated with new-onset heart failure in a cohort study of members of the general population (43). FGF23 may induce HF through FGF23-associated LVH, and Andersen et al. reported that the circulating concentrations of FGF23 in patients with HF are significantly higher than those in healthy individuals (50). In this study, the expression of FGF23 in the ventricles of patients with HF did not exceed those of heathy controls (50), but several other studies have shown higher expression of FGF23 in the myocardia of patients with LVH and in rodent models of LVH (11–14). Genetically high FGF23 concentrations have also been shown to be associated with a higher risk of heart failure in a biobank cohort (51). Thus, the circulating concentration of FGF23 and its myocardial expression may be associated with HF.

### 2.3 FGF23 and atrial fibrillation

Numerous studies have shown a relationship between FGF23 concentration and atrial fibrillation (AF) (23, 39, 47, 52–55). The Multi-Ethnic Study of Atherosclerosis (MESA) and Cardiovascular Health Study (CHS) revealed that high serum concentrations of FGF23 are associated with the incidence of AF, even after adjustment for estimated glomerular filtration rate and other cardiovascular risk factors (52, 53). In contrast, the Atherosclerosis Risk in Communities (ARIC) study showed that the baseline serum FGF23 concentration is not associated with the risk of AF after adjustment for potential confounders (52). However, a meta-analysis showed that high concentrations of FGF23 are associated with a higher risk of AF (52). Recently, Graves et al. demonstrated that FGF23 prolongs the QTc interval and induces ventricular arrhythmias *via* the FGFR4 pathway in mice (56). It is thought that FGF23 induces LVH, leading to cardiac remodeling, which may explain the arrhythmogenesis.

### 2.4 FGF23 and myocardial infarction

Some previous studies have demonstrated that FGF23 concentration is associated with the incidence of myocardial infarction (MI) (22, 37), whereas others have shown no association (35, 57, 58). Thus, this remains an area of controversy, but the principal effect of FGF23 on the heart is likely to be the induction of LVH, which may exacerbate HF and AF.

### 2.5 FGF23 and cardiovascular mortality

Several previous studies have shown an association between high serum concentrations of FGF23 and cardiovascular mortality (37, 45, 59–64). Furthermore, the circulating concentrations of FGF23 are significantly higher in non-surviving patients with myocardial infarction and heart failure (65). A linear dose-response relationship between FGF23 concentration and cardiovascular mortality for concentrations of FGF23 of >50 pg/mL has been demonstrated (37, 59). FGF23 concentrations are stable over time in the majority of patients with CKD; however, individuals with rising FGF23 concentrations were shown to be at a higher risk of death than those with stable FGF23 concentrations (66). These findings imply that high FGF23 concentration is associated with cardiovascular mortality owing to HF and AF.

## 3 Mechanisms of FGF23-induced LVH

Numerous studies have demonstrated that high FGF23 concentrations are associated with LVH in humans (21–25, 27–30). In 2011, Faul et al. demonstrated experimentally that the intramyocardial injection of FGF23 in mice induces LVH (26). Cardiomyocytes express FGFR4, but α-klotho is not expressed in the heart. α-klotho increases the binding affinity of FGFR to FGF23 by ~20-fold (67); therefore, the binding affinity of FGF23 for FGFR4 in the absence of α-klotho is weaker than in its presence. Thus, both direct and indirect mechanisms of the effect of FGF23 on the heart must be discussed to fully understand how FGF23 influences the progression of LVH.

### 3.1 Mechanism for the direct effect of FGF23 on LVH

Faul and colleagues have demonstrated that the injection of recombinant FGF23 induces LVH in an FGFR-dependent, but α-klotho-independent, manner (26). They and other researchers have shown that FGF23 increases the expression of pro-hypertrophic genes in cardiomyocytes (12, 26, 68), regulates calcium homeostasis in cardiomyocytes (69), increases intracellular calcium concentration, and promotes the contractility of cardiomyocytes (70) (**Table 1**). FGF23 activates FGFR4/phospholipase Cγ/calcineurin/nuclear factor of activated T-cells (NFAT) signaling in cardiomyocytes and induces cardiac hypertrophy in rodents (71). In addition, Han et al. have shown that the cardiac-specific deletion of FGFR4 attenuates FGF23-induced LVH in mice (74). These results are consistent with FGF23 directly stimulating cardiomyocytes *via* FGFR4 to induce LVH. Klotho-independent signaling is only activated in the presence of a high FGF23 concentration (67). The upregulation of intracardiac FGF23 expression using an adeno-associated virus (AAV) was found not to induce LVH in healthy mice (75). In addition, high-phosphate diet-induced LVH in mice, which is mediated through high serum FGF23 concentrations, was found to be reversed by the normalization of the serum FGF23 concentration (72). Therefore, this direct effect of FGF23 on the heart is likely to occur only under pathological conditions, such as in a CKD-related milieu.

**Table 1:** Summary of the results of experimental studies regarding the direct effects of FGF23 on LVH and myocardial fibrosis.

Author	Year	Cell or animal	Effects of FGF23	Reference
**Faul et al**	2011	NRVMs C57BL/6 mice	increase of hypertrophic genes increase of cell surface area increase of LVH	(26)
**Touchberry et al**	2013	HL-1 cardiomyocytes Mouse ventricular tissue Mouse primary cardiomyocyte	increase of cell surface area increase of hypertrophic genes increase of intracellular calcium and contractile force	(70)
**Grabner et al**	2015	NRVMs	increase of hypertrophic genes activation of FGFR4 /phospholipase Cγ / calcineurin / NFAT signaling	(71)
**Huang et al**	2016	Rabbit cardiomyocytes	increase of intracellular calcium, beat rates and mitochondrial ROS	(69)
**Hao et al**	2016	AMCFa C57BL/6 mice with ligation of left coronary artery or ischemia / reperfusion	increase of fibrosis-related genes increase of myocardial fibrosis	(10)
**Leifheit-Nestler et al**	2017	NRVMs	increase of cell surface area	(12)
**Grabner et al**	2017	NRVMs	increase of cell surface area, and reverse to normal size after FGF23 removal	(72)
**Mhatre et al**	2018	NRVMs	increase of cell surface area	(68)
**Leifheit-Nestler et al**	2018	NRVMs NRCFs	increase of hypertrophic genes increase of fibrosis-related genes	(73)
**Han et al**	2020	C57BL/6 mice Cardiac-myocyte specific loss of FGFR4 mice	increase of LVH prevention of FGF23-induced LVH	(74)
**Leifheit-Nestler et al**	2021	NRVMs C57BL/6 mice	increase of hypertrophic genes induced by AAV-fgf23 no LVH in healthy mice with AAV-fgf23	(75)
**Eitner et al**	2022	myocyte specific loss of FGF23 mice	a more severe reduced cardiac function increased expression of FGF23 in cardiac fibroblasts and endothelial cells	(76)
**Lee et al**	2022	Human atrial fibroblast	migration and proliferation	(77)

NRVMs, Neonatal rat ventricular cardiomyocytes; NFAT, nuclear factor of activated T cell; ROS, reactive oxygen species; AMCFs, adult mouse cardiac fibroblasts; NRCFs, neonatal rat cardiac fibroblasts; AAV, adeno-associated virus.

**Figure 1:**
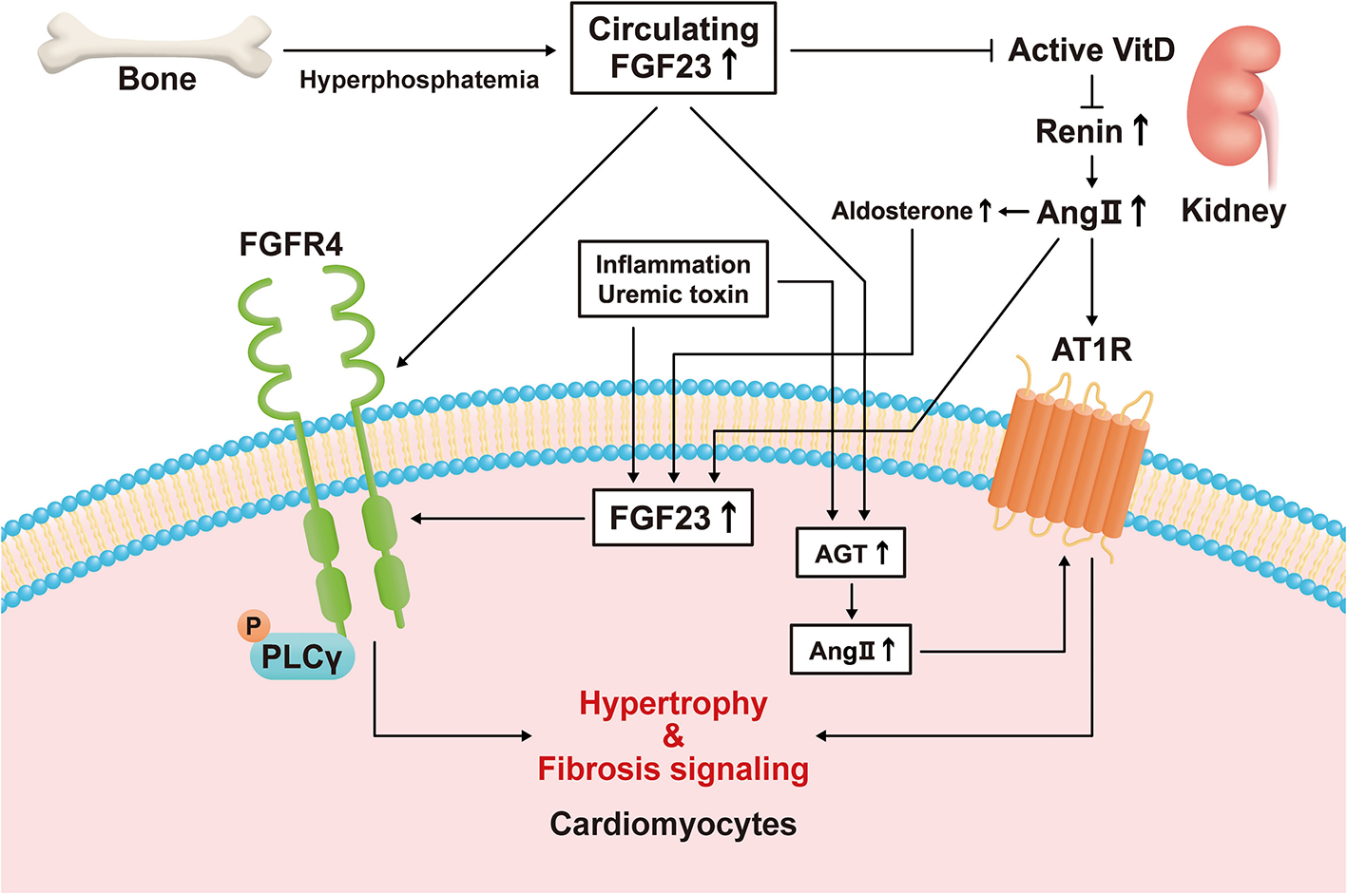
Direct and indirect mechanisms mediate the effects of fibroblast growth factor (FGF)23 on left ventricular hypertrophy. Hyperphosphatemia induces an increase in circulating FGF23 concentration by increasing its secretion by bone. FGF23 stimulates hypertrophic signaling *via* fibroblast growth factor receptor (FGFR)4 in cardiomyocytes. FGF23 also suppresses active vitamin D (VitD) synthesis in the kidney, and the activation of VitD is lower in a chronic kidney disease (CKD)-related milieu. Active VitD inhibits renin activity in the kidney and heart and increases serum angiotensin II (Ang II) concentration and its cardiac expression. Ang II binds to angiotensin II receptor type 1 (AT1R) in cardiomyocytes, causing cardiac hypertrophy and fibrosis. Inflammatory cytokines, a uremic milieu, Ang II, and aldosterone induce FGF23 transcription in cardiomyocytes. Circulating FGF23 also causes an increase in local angiotensinogen and Ang II expression in cardiomyocytes, leading to hypertrophy and fibrosis.

Several previous studies have demonstrated that the expression of FGF23 in cardiomyocytes is high under pathological conditions. The expression of FGF23 and FGFR4 in the heart has been shown to be associated with LVH using autopsy samples collected from patients with CKD (11), and the expression of FGF23 is high in the heart following MI (9). Transverse aortic constriction (TAC)-induced LVH causes an increase in the expression of FGF23 in the heart (10, 13, 14). In addition, rats that undergo nephrectomy express FGF23 in their hearts (12). Inflammation regulates the expression of FGF23 through HIF1α stabilization in osteocytes (78). Finally, the uremic toxin indoxyl sulfate induces cardiac hypertrophy through the FGF23-FGFR4 signaling pathway (79). These results suggest that inflammation caused by HF or uremic toxins may induce the expression of FGF23 in cardiomyocytes (**Figure 1**).

Previous studies have also shown that FGFR4 mediates LVH (71, 72, 74). FGFR4 knockout mice attenuated the progression of LVH which was induced by high phosphate diet, FGF23, and aging (71, 72, 74). The expression of FGFR4 in the heart changes under pathological conditions. Hao et al. showed that the expression of FGFR4 increases in the heart after ischemic reperfusion (10), and cardiac FGFR4 expression was shown to be markedly upregulated in the hearts of patients with LVH (11). Finally, cardiac overexpression of FGF23 using an adeno-associated virus (AAV) in mice was found to increase the expression of FGFR4 (75). These findings imply that the level of FGFR4 expression in the heart may contribute to the progression of LVH.

FGF23 has also been reported to induce myocardial fibrosis. Hao et al. demonstrated that it is expressed in cardiac fibroblasts and its overexpression in the heart induces cardiac fibrosis through the activation of β-catenin and TGF-β in mice (10). FGF23 induces pro-fibrotic signaling, involving TGFβ/Smad complexes, in cardiac fibroblasts (73, 80). In addition, FGFR1 has been reported to contribute to the FGF23-induced proliferation and migration of cardiac fibroblasts (77). Recently, Eitner et al. demonstrated that the myocyte-specific deletion of FGF23 mice with TAC-induced LVH impairs cardiac function and is associated with higher expression of FGF23 in fibroblasts and endothelial cells (76). These findings suggest that cardiac myocyte-derived FGF23 is needed to maintain cardiac function and that cardiac fibroblasts and endothelial cells might represent important sources of FGF23 for the progression of LVH under pathological conditions. Together, these results are consistent with the hypothesis that FGF23/FGFR induces fibrotic signaling in cardiac fibroblasts.

Previous studies have shown that FGF23 has effects on other types of cardiovascular cells. High serum FGF23 concentrations are associated with endothelial dysfunction in patients with CKD (81), and FGF23 has been shown to cause the release of nitric oxide (NO) and the formation of reactive oxygen species (ROS) in human coronary artery endothelial cells (82). Pro-inflammatory M1 macrophages express FGF23 (17), and FGF23 has been shown to increase the production of the pro-inflammatory cytokine TNF-α by M0 macrophages and to reduce arginase-1 expression in M2 macrophages (17). Thus, FGF23 can be expressed in many cell types, including myocytes, fibroblasts, endothelial cells, and cardiac macrophages under pathological conditions, and the paracrine effects of FGF23 secreted by these cells, in addition to the effects of circulating FGF23, may influence pathological cardiac remodeling (83).

### 3.2 Mechanism of the indirect effect of FGF23 on LVH

There are several hypotheses regarding how an indirect mechanism might mediate the effect of FGF23 on LVH (**Figure 1** and **Table 2**). Slavic et al. reported that TAC increases the circulating FGF23 concentration and the cardiac expression of FGF23 in mice (13). Okamoto et al. also demonstrated that the LVH induced by TAC is associated with high cardiac FGF23 expression and RAAS activation (88). However, genetic ablation of *Fgf23* does not affect TAC-induced LVH and spironolactone inhibits LVH following TAC (13). Leifheit-Nestler et al. showed that both angiotensin II and aldosterone induce FGF23 expression in cardiomyocytes (73), and Mhatre et al. showed that both FGF23 and angiotensin II stimulate an increase in cytoplasmic Ca^2+^ in cardiomyocytes and induce LVH (68). Finally, Böckmann et al. revealed that FGF23 induces the expression of angiotensinogen gene in cardiomyocytes and angiotensin-converting enzyme in cardiac fibroblasts, activates the cardiac RAAS, and promotes LVH (85). Thus, the RAAS plays an important role in the development of LVH, and FGF23 is associated with cardiac RAAS activation in LVH.

**Table 2:** Summary of the results of experimental studies regarding the indirect effects of FGF23 on LVH and myocardial fibrosis.

Author	Year	Cell or animal	Effects	Reference
**Andrukhova et al**	2014	FGF23 knock out mice	FGF23 increases the expression of NCC and induces high blood pressure and LVH	(84)
**Leifheit-Nestler et al**	2017	Sprague Dawley rats with 5/6 nephrectomy NRVMs	Calcitriol attenuates cardiac FGF23/FGFR4 and hypertrophy Calcitriol inhibits FGF23-mediated hypertrophic growth	(12)
**Mhatre et al**	2018	NRVMs	FGF23 mediates cardiac hypertrophy via AngIIexpression	(68)
**Bockmann et al**	2019	NRVMs and NRCFs	FGF23 stimulate RAAS genes and mireralocorticoid receptor activation	(85)
**Czaya et al**	2019	Sprague Dawley rats with 5/6 nephrectomy	Paricalcitol and pan-FGFR blocker suppresses LVH	(86)
**Inoue et al**	2021	Wister rats with heminephrectomy NRVMs	Maxacalcitol retards AngII induced- LVH by inhibition of calcineurin-NFAT activity Maxacalcitol suppresses AngII induced calcineurin-NFAT activity	(87)
**Okamoto et al**	2022	C57BL/6 mice with TAC induced LVH	LVH is associated with expression of FGF23 and RAAS activation	(88)
**Saito et al**	2023	Deoxycorticosterone cetae-salt mice	Calcitriol attenuates FGF23-induced cardiac fibrosis	(89)

NRVMs, neonatal rat ventricular cardiomyocytes; AngII, angiotensin II; NRCFs, neonatal rat cardiac fibroblasts; TAC, transverse aortic constriction; RAAS, renin-angiotensin-aldosterone system; NCC, sodium chloride cotransporter.

Active vitamin D inhibits RAAS-associated gene expression and reduces cardiac fibrosis (90–92). It also increases the serum FGF23 concentration, but inhibits FGF23-FGFR4 signaling in the heart and reduces LVH (12). Active vitamin D has been shown to retard the progression of LVH by inhibiting calcineurin/NFAT activity (87). In addition, active vitamin D and a pan-FGFR blocker have additive effects to further slow LVH (86). Recently, Saito et al. have shown that active vitamin D attenuates FGF23-induced cardiac fibrosis and improves diastolic function by inhibiting TGF-β signaling in deoxycorticosterone acetate and salt-treated mice (89). Finally, FGF23 reduces the synthesis of active vitamin D in the kidney (7). Thus, the downregulation of active vitamin D secondary to a high serum FGF23 concentration may contribute to the progression of LVH.

One previous study showed an effect of FGF23 on the sodium chloride co-transporter NCC. Specifically, FGF23 directly increased the expression of NCC in the distal renal tubules and sodium reabsorption in mice (84). This result suggests that FGF23 might induce volume expansion and high blood pressure through the upregulation of NCC, thereby contributing to LVH.

## 4 Conclusion

FGF23 contributes to the progression of LVH through direct and indirect mechanisms. However, the progression of LVH is also affected by active vitamin D, the RAAS, blood pressure, and other factors. In a CKD-related milieu in particular, high expression of FGF23 in osteocytes and cardiomyocytes may contribute to LVH progression *via* FGFR4 and angiotensin II receptor type 1 signaling.

## Author contributions

TN contributed to provide the concept of review and drafting the manuscript. HK contributed to critical revision of the manuscript. MT contributed to provide the concept of review and contributed to critical revision of the manuscript. All authors contributed to the article and approved the submitted version.
